# Locomotor-Like Leg Movements Evoked by Rhythmic Arm Movements in Humans

**DOI:** 10.1371/journal.pone.0090775

**Published:** 2014-03-07

**Authors:** Francesca Sylos-Labini, Yuri P. Ivanenko, Michael J. MacLellan, Germana Cappellini, Richard E. Poppele, Francesco Lacquaniti

**Affiliations:** 1 Laboratory of Neuromotor Physiology, Santa Lucia Foundation, Rome, Italy; 2 Centre of Space Bio-medicine, University of Rome Tor Vergata, Rome, Italy; 3 Louisiana State University, School of Kinesiology, Baton Rouge, Louisiana, United States of America; 4 Department of Neuroscience, University of Minnesota, Minneapolis, Minnesota, United States of America; 5 Department of Systems Medicine, University of Rome Tor Vergata, Rome, Italy; VU University Amsterdam, Netherlands

## Abstract

Motion of the upper limbs is often coupled to that of the lower limbs in human bipedal locomotion. It is unclear, however, whether the functional coupling between upper and lower limbs is bi-directional, i.e. whether arm movements can affect the lumbosacral locomotor circuitry. Here we tested the effects of voluntary rhythmic arm movements on the lower limbs. Participants lay horizontally on their side with each leg suspended in an unloading exoskeleton. They moved their arms on an overhead treadmill as if they walked on their hands. Hand-walking in the antero-posterior direction resulted in significant locomotor-like movements of the legs in 58% of the participants. We further investigated quantitatively the responses in a subset of the responsive subjects. We found that the electromyographic (EMG) activity of proximal leg muscles was modulated over each cycle with a timing similar to that of normal locomotion. The frequency of kinematic and EMG oscillations in the legs typically differed from that of arm oscillations. The effect of hand-walking was direction specific since medio-lateral arm movements did not evoke appreciably leg air-stepping. Using externally imposed trunk movements and biomechanical modelling, we ruled out that the leg movements associated with hand-walking were mainly due to the mechanical transmission of trunk oscillations. EMG activity in hamstring muscles associated with hand-walking often continued when the leg movements were transiently blocked by the experimenter or following the termination of arm movements. The present results reinforce the idea that there exists a functional neural coupling between arm and legs.

## Introduction

Humans swing the arms in an automatic, stereotypical way during locomotion by pairing upper and lower limb movements with integer ratio frequencies, as do animals during quadrupedal locomotion [1]–[4]. These arm movements are characteristic of walking, running, crawling, swimming, climbing and other gaits, but they are not obligatory (as when we walk with crossed arms). Arm movements during locomotion might result from passive mechanical coupling with trunk and shoulder movements, as well as from active motor strategies aimed at reducing overall energy expenditure [1], [5], [6] or enhancing gait stability [7], [8]. Rhythmical activity of arm and shoulder muscles is present during upright walking [9]–[12] even when the arms are immobilized during walking [9], [13], indicating the influence of a central motor program. The coordination between arms and legs during human locomotion shares many features with that in quadrupeds, including the reliance on propriospinal connections [2], [14]–[19]. In animals, inter-limb coordination may also reflect supraspinal control; thus, hindlimb-related neurons in cat motor cortex respond to changes in forelimb movements during locomotion [20].

Inter-limb coupling in humans has previously been studied by evoking reflexes in one limb and observing the extent to which the movement of another limb modulates reflex expression [21]–[24]. Recently, it has been shown that active arm movements increase leg muscle recruitment during sub-maximal recumbent stepping [25]. It has been argued that a better understanding of the mechanisms underlying the coordination of the four limbs might bear important implications for locomotor rehabilitation in several neuromotor disorders [22], [25]–[27].

In quadrupeds, forelimb movements may facilitate or even trigger hindlimb stepping, consistent with a coupling between cervical and lumbosacral central pattern generators (CPG) [19], [28]–[31]. It is less clear, however, whether such a facilitation can also be shown in humans, who presumably have a weaker coupling between upper and lower limbs movements in relation to the evolution of bipedal locomotion and the need to free the upper limbs for manipulative tasks. Here we tried to reveal facilitation of lower limb stepping by upper limb movements by asking participants to move their arms overhead rhythmically, as in hand-walking. We hypothesized that these arm movements might trigger automatic, alternating movements of the legs, evocative of locomotor-like patterns. We used an air-stepping protocol, because it has been shown that, at least in the case of tonic sensory stimulation or spinal electromagnetic stimulation, automatic leg movements are easier to evoke in the absence of limb loading and balance control [32]–[35].

## Methods

### Participants

In a first series of experiments, we screened 33 healthy volunteers naïve to the purpose of the experiments (age range 23–50 yrs, 18 males and 15 females, leg length 0.83±0.04 m [mean±SD], height 1.72±0.10 m, weight 68±7 kg) for the presence of leg responses. Nine of these subjects (age range 25–45 yrs, 4 males and 5 females, mean leg length 0.86±0.05 m, height 1.73±0.10 m, weight 69±10 kg), in whom prominent leg movements could be elicited by arm movements and who were able to return to the laboratory to participate in additional sessions, were selected for detailed kinematic and EMG recordings in a second series of experiments. The studies conformed to the Declaration of Helsinki, and we obtained informed consent from all the participants according to the procedures of the Ethics Committee of the Santa Lucia Foundation.

### Experimental set-up

**Figure 1:**
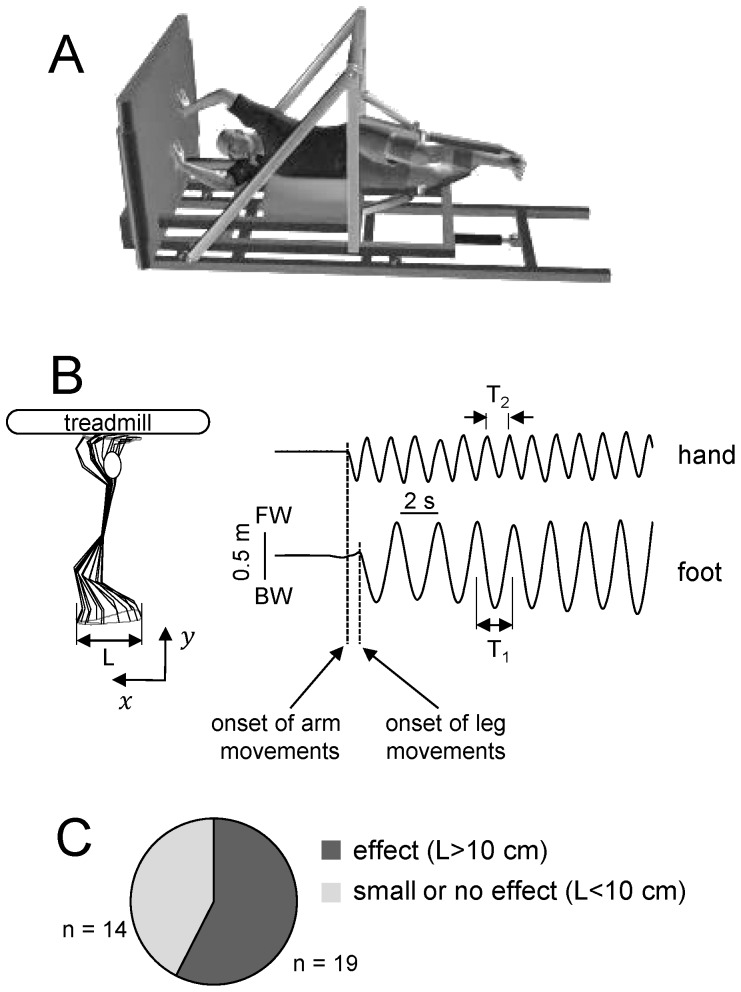
Experimental setup. A – schematic illustration of the horizontal body weight support system. The subject lay on the right side with each leg suspended in an exoskeleton allowing low-friction segment movements. The arms were unrestricted and unsupported. The head rested on a small pillow placed on a 10-cm wide horizontal belt (not shown) in such a way that the lower arm moved unimpededly. B – an example of foot (MP marker) anterior–posterior displacement evoked by hand-walking (represented by DP marker displacement). FW – forward, BW – backward. L indicates foot excursion. Vertical dashed lines indicate the onset of arm and leg movements. Note the difference between the foot and hand cycle periods (T_1_ and T_2_, respectively). C – pie chart showing the percentage of subjects (n = 33) in which hand-walking evoked significant (L>10 cm) foot displacements and of subjects with small or no effect (L<10 cm).

We reduced the effective forces due to gravity in the plane of movement of the lower limbs using an exoskeleton placed horizontally (Italian patent #Rm2007A000489). The system has been described in detail elsewhere [35]–[37]. Briefly, the subject lay on the right side with each leg suspended in an independent exoskeleton, allowing low-friction, low-inertia segment movements (Fig. 1A). The system neutralizes the component of the gravity force normal to the lying surface. The length of the telescopic thigh segment of the exoskeleton was adjusted according to the length of the subject's thigh, and the leg was attached by means of a cuff to the exoskeleton, in such a manner as to provide the best alignment of the axes of rotation of the hip and knee joints with those of the exoskeleton. The foot segment remained unrestrained. In order to provide a comfortable step width, we also adjusted the angle between the two legs by tilting the structure which supported the upper part of the exoskeleton relative to the couch. The upper body of the subject was secured through a chest and shoulder fixation, while the head rested on a pillow. Even though anterior-posterior trunk movements were limited, the hip support could slide along the anterior-posterior tracks of the couch, thus allowing pelvis rotation. A treadmill (EN-Mill 3446.527, Bonte Zwolle BV, The Netherlands) was tilted by 90° and placed at about arm-length distance from the subject's shoulders, orthogonal to his/her body. The comfortable distance (long enough to provide sufficient shoulder and elbow flexion/extension but not too far to permit an easy contact with the treadmill) from the shoulder to the treadmill belt was about 80% of the total upper limb length (forearm+upper arm) in all conditions. In the main series of experiments, the treadmill belt moved in the sagittal (antero-posterior and posterior-anterior) direction relative to the lying subject (Fig. 1A). In an additional experiment, the treadmill was rotated so that its belt moved in the frontal (medio-lateral) direction relative to the subject.

### Protocol

We asked participants to reach overhead to the treadmill and rhythmically displace their hands on the shifting belt of the treadmill, as if they walked on the hands. The arms were unrestricted and unsupported (the head rested on a small pillow placed on a 10-cm wide horizontal belt in such a way that the lower arm moved unimpededly). To ‘hand-walk’ on the treadmill belt the participants needed to reach to contact the surface and actively extend the arm. To reduce the attention level paid to the locomotor task, subjects were also asked to carry out mental arithmetic (counting down out aloud by 7′s) throughout each trial [33]. We did not provide any instruction concerning the posture to be maintained with the body and lower limbs. By varying treadmill speed in different trials, we were able to change the speed of arm movements accordingly. The treadmill belt could shift in either anterior-posterior (a-p), posterior-anterior (p-a) or medio-lateral (m-l) directions in different trials. The protocol and instruction were the following. First, the subject was asked to extend both arms overhead without touching the treadmill (initial position). In about 2–3 s, the treadmill belt began to move at a given speed and the recording started. Then (in about 3–5 s), the experimenter told the subject a random number and he/she started to count and ‘hand-walk’ on the treadmill. The duration of each trial was ∼1–2 min, with at least 2-min rest between trials. At the end of the trial, the experimenter told the subject to stop counting and tuned off the treadmill belt movement. Hand-walking termination was not recorded in the main protocol (Table 1).

**Table 1:** Experimental conditions.

	main protocol				additional experiments			
	a-p hand-walking	p-a hand-walking	a-p arm air-stepping	vol. leg air-stepping	m-l hand-walking	hand-walking termination	transient leg block	passive hip disp.
	0.5–4 km/h	2 km/h			1 km/h	1 km/h	1–3 km/h	
s1	x	x	x	x	-	-	-	x
s2	x	x	x	x	-	-	x	x
s3	x	x	x	x	-	-	-	-
s4	x	x	x	x	-	-	-	-
s5	x	x	x	x	x	x	x	-
s6	x	x	x	x	x	x	x	x
s7	x	x	x	x	-	-	x	x
s8	x	x	x	x	x	x	x	x
s9	x	x	x	x	x	x	x	-

All participants (n = 33) were initially tested for the presence of automatic leg movements induced by hand-walking at 1 and 2 km/h in both a-p and p-a directions. After completing the test, we asked the subjects whether they had noticed an appearance of leg movements during hand-walking. These sessions were video-recorded, without automatic motion capture and EMG data collection, mean foot excursion was approximately assessed using a ruler (on average 28±24 cm, range 3–85 cm).

Next, we collected detailed kinematic and EMG data in 2 additional sessions (Table 1), carried out on different days, in 9 subjects among those who demonstrated prominent automatic movements of the legs (foot excursion >20 cm) in response to hand-walking during initial testing. In one session, the participants hand-walked on the treadmill at different speeds in the a-p direction (0.5, 1, 2, 3 and 4 km/h, in random order), and in the p-a direction at 2 km/h. In the final part of this session, two additional tests were conducted. In the first test, we asked the subjects to generate self-paced stepping-like arm movements in air in the a-p direction and, in the second test, we asked the subjects to perform leg movements voluntarily for 20–30 s (without arm movements) while lying in the apparatus as before. The total duration of this experimental session was ∼2 h.

In another session, four supplementary experiments were performed on subsets of subjects (Table 1). In one experiment, we asked the subjects to perform hand-walking at 1 km/h in the medio-lateral (m-l) direction. To this end, the treadmill was placed perpendicular to the ground floor with the belt moving in the bottom-up direction corresponding to rightward hand-walking. In another experiment, we also recorded leg movements when hand-walking terminated (three trials at 1 km/h in the a-p direction for each subject): the participants were instructed to stop hand-walking (maintaining a stationary arm position), when the treadmill was arrested, but to continue counting. In the third experiment, we tested the effect of a transient block of leg stepping movements evoked by hand-walking. To this end, an experimenter (placed behind the subject, unseen to him/her) blocked for several seconds the subject's legs at about the central position of their excursion during hand-walking in the a-p direction at 1, 2 and 3 km/h (randomly selected speeds, 42 trials for 6 subjects). The block was obtained by manually holding both shank segments of the exoskeleton firmly. In the fourth experiment, we tested the potential mechanical effects of hip displacements on leg movements. To this end, an experimenter manually displaced the lower trunk of the subject back and forth by ∼5 cm (comparable to the average amplitude of trunk displacements measured during hand-walking in the previous session, see Results) while the subject remained passive.

### Data recording

We recorded kinematic data bilaterally at 100 Hz by means of the Vicon-612 system (Oxford, UK) with nine cameras spaced around the system. Infrared reflective markers (diameter 1.4 cm) were attached on each side of the subject to the skin overlying the following landmarks: the end of 3rd distal phalanx (DP) of the hand, the wrist, elbow, gleno-humeral joint, greater trochanter (GT), lateral femur epicondyle (LE), lateral malleolus, heel, and fifth metatarso-phalangeal joint (MP). The GT marker of the right side of the body could not be recorded (because the subject lay on this side). 20-cm sticks with two markers were attached to GT and LE of the left leg, and the GT and LE positions were reconstructed as the midpoint between these two markers.

EMG activity was recorded bilaterally by means of surface electrodes from 13 muscles simultaneously: flexor carpi ulnaris (FCU), extensor carpi ulnaris (ECU), long head of triceps brachii (TRIC), long head of biceps brachii (BIC), anterior deltoid (DELTa), posterior deltoid (DELTp), semitendinosus (ST), biceps femoris (BF), vastus medialis (Vmed), rectus femoris (RF), tibialis anterior (TA), gastrocnemius medialis (MG) and soleus (SOL). The EMG data were recorded with the wireless Delsys Trigno EMG system (Delsys Inc., Boston, MA), bandwidth of 20–450 Hz, overall gain of 1000, and digitized at 2000 Hz. Sampling of kinematic and EMG data was synchronized.

### Data analysis

Gait cycle was defined independently for arm and leg movements as the time interval between two successive maxima of the a-p displacement of the DP marker for the arms and the MP marker for the legs (T_1_ and T_2_, respectively, in Fig. 1B). Amplitudes of shoulder, elbow, wrist, hip, knee, and ankle joint angular changes were computed [17] and averaged across ∼10 cycles during the last 15–30 s of each trial. The direction of evoked leg air-stepping (forward or backward) was evaluated by calculating the signed area of foot (MP marker) trajectory over the cycle (normalized to the foot excursion) and averaged across all cycles in the trial:
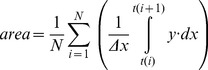
(1)where *x* and y are the coordinates of the MP marker (Fig. 1B), *Δx* is the foot a-p excursion in the i-th cycle, *t(i)* and *t(i+1)* are the onsets of the i and i+1 leg cycles, respectively, and *N* is the number of cycles in the trial. According to this criterion, the movement was considered forward if the area was positive and backward if the area was negative.

To evaluate the percentage of subjects in the original sample of 33 participants in whom hand-walking evoked significant foot displacements, we set a threshold of 10 cm (L) based on the amplitude (peak-to-peak) of foot (MP marker) displacements caused by passive hip movements (see *Effects of trunk oscillations on leg motion* in the Results). We therefore considered lower limb oscillations with L<10 cm as possibly due to a mechanical effect of hand movements on leg motion.

**Figure 2:**
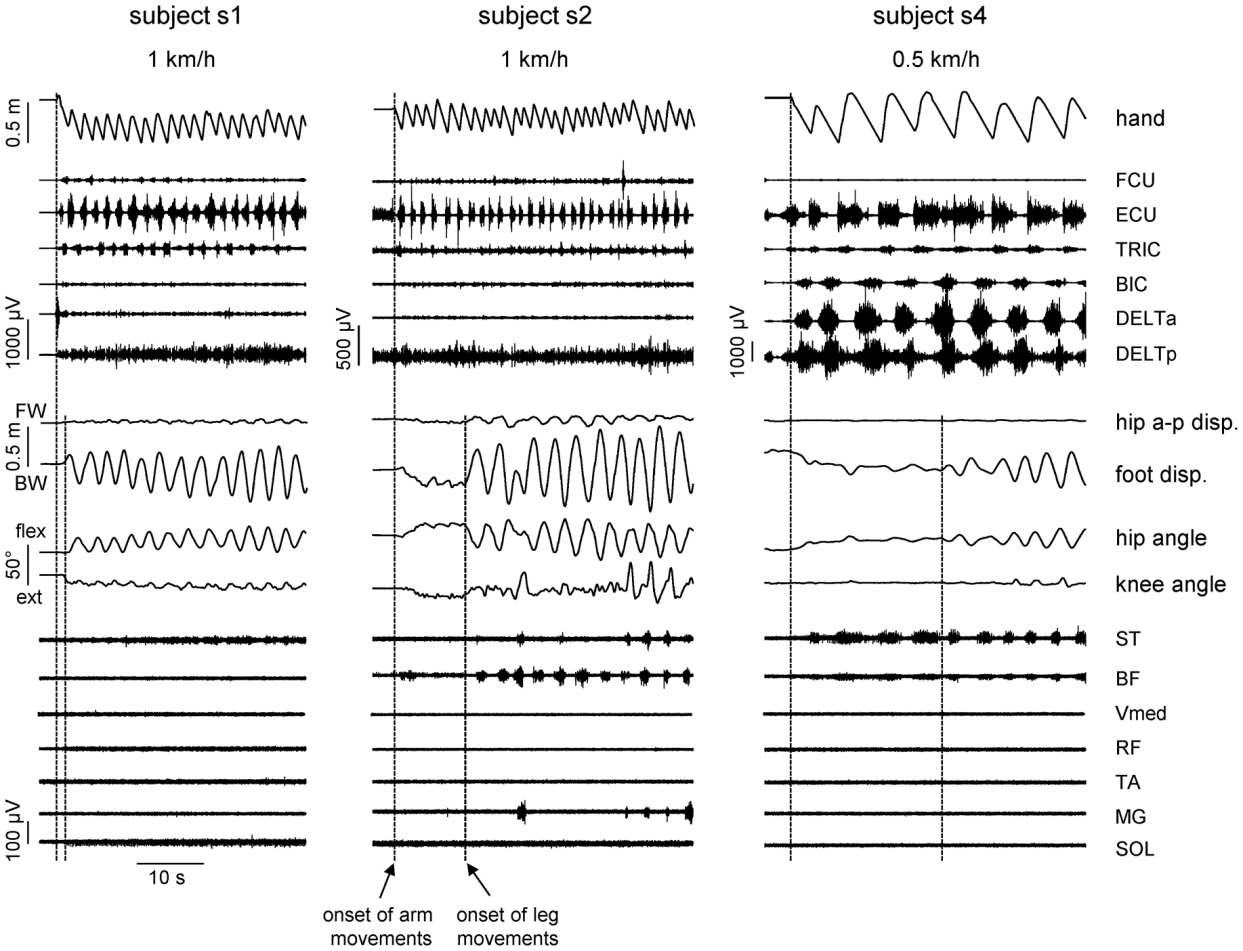
Stepping initiation. Examples of the delay of evoked air stepping from the onset of arm movements (at speeds indicated on the top of the figure) in three different subjects. From *top* to *bottom*: anterior-posterior (a-p) displacement (disp.) of the left hand, EMG activity of 6 muscles of the left arm, left hip and foot a-p displacements, left hip and knee joint angles and EMG activity of 7 muscles of the left leg. Vertical dashed lines indicate the onset of arm and leg movements. FCU, flexor carpi ulnaris; ECU, extensor carpi ulnaris; TRIC, long head of triceps brachii; BIC, long head of biceps brachii; DELTa, anterior deltoid; DELTp, posterior deltoid; ST, semitendinosus; BF, biceps femoris; Vmed, vastus medialis; RF, rectus femoris; TA, tibialis anterior; MG gastrocnemius medialis; SOL soleus. Note variability in the onset of leg stepping across the subjects.

We determined the onset of leg motion as the last critical point prior to the first rhythmic cycle with L>10 cm where the time derivative of foot (MP marker) excursion changed its sign (Fig. 2). The onset of arm motion was defined when the hand end-point excursion exceeded 2 standard deviations from the mean value calculated during the rest period at the beginning of each trial, because there were always small oscillations of the hand end-point due to the unsupported initial arm position (see Protocol).

The EMG signals were numerically rectified, low-pass filtered using a zero-lag 4^th^-order Butterworth filter with a cut-off of 10 Hz. Kinematic and EMG data were time-interpolated over individual gait cycles to fit a normalized 100-point time base and averaged across (∼10) cycles. For averaging across subjects, we used both non-normalized (in µV) and normalized (to the maximum value) EMG data. For the normalized method, EMG envelopes were assumed to be zero if the maximum value was less than 3 µV (that we considered to be the noise level). We also analysed limb kinematics and EMG signals in the frequency domain by using a fast Fourier transform (‘fft.m’ function in Matlab). We computed the frequency component with the highest amplitude (peak-frequency), its phase shift relative to the onset of the leg cycle (zero corresponds to the cosine function with zero time shift) and the percent of variance (r^2^) accounted for by this component.

When the ratio between arm and leg cycle durations was ∼1 (in the range 0.95÷1.05), ipsilateral phase lag (*IPL*) between upper and lower limbs was determined using the methods described previously [17]. In brief, the relative timing of left lower limb cycle onset was expressed as a percentage of the gait cycle determined by consecutive left hand contacts:
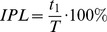
(2)where t_1_ is interval of time between left leg cycle onset and left hand touchdown events and T is the hand cycle duration. According to this method, lateral gait patterns (ipsilateral upper/lower limb contact at similar instances) are determined at a value of 0% and diagonal gait patterns (contralateral upper/lower limb contact at similar instances) are determined at a value of 50%. Intermediate values (∼25%) correspond to no limb pairing.

To evaluate the performance of mental arithmetic, an experimenter annotated all the numbers pronounced throughout the trials in four subjects. For each trial, we calculated the percent of errors as the amount of wrong numbers divided by the total amount of numbers and multiplied by 100, and the rate of counting as the total amount of numbers divided by the duration of the trial (in minutes).

### Modeling the mechanical effects of hip motion on leg movements

We considered the potential mechanical effects of trunk oscillations on leg motion using both biomechanical modeling and the effect of externally imposed trunk movements as assessed in the additional experiments (Table 1). In particular, based on these two approaches, we identified *a posteriori* the lower threshold of foot excursions used to calculate the percent of “responsive” subjects from the initial screening experiments (see Results).

**Figure 3:**
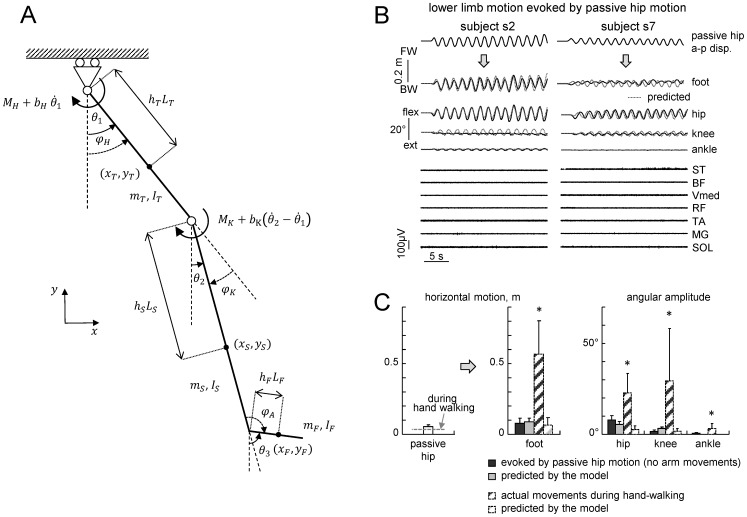
Biomechanical model. A – schematic representation of the biomechanical model used to estimate the mechanical effect of periodic a-p hip displacements on leg motion. B – examples of passive lower limb motion evoked by relatively large (10 and 5 cm) hip oscillations in two subjects. The upper curves represent passive hip displacements manually induced by the experimenter while the lower curves represent the resulting leg movements (foot a-p displacements and joint angles) and leg muscle EMGs. Dotted lines represent the prediction made using the biomechanical model. Note the absence of EMG activity during lower limb movements evoked by passive hip motion. C – mean (+SD) horizontal foot and joint angular excursion (peak-to-peak) evoked by passive hip motion and by hand-walking (mean for all treadmill speeds) as well as predicted by the model. Note significantly smaller foot displacements evoked by passive hip motion or estimated from the model (L<10 cm) relative to those during hand-walking (∼60 cm). Asterisks denote significant differences (p<0.05) with Tukey HSD multiple comparison tests.

A biomechanical model was used to simulate the purely mechanical effects of trunk oscillations on leg movements. The lower limb was modeled as a multi-pendulum with three rigid, homogeneous segments (Fig. 3A): thigh, shank and foot, with mass *m_T_*, *m_S_* and *m_F_*, length *L_T_*, *L_S_* and *L_F_*, and moment of inertia *I_T_*, *I_S_* and *I_F_*, respectively. Hip and knee joints were modeled as frictionless hinges with linear dampers, damping coefficients being *b_H_* and *b_K_* respectively [38]. Moreover, to account for passive elastic coupling due to mono- and bi-articular muscles, for each joint we considered a passive elastic joint moment (*M_H_* and *M_K_*) as a function of lower limb joint angles, following the model of Riener and Edrich [39]. We considered the ankle joint fixed, because we did not observe any significant angular motion at this joint during the pertinent experiments (see Results).

The coordinates of the center of mass of each segment are:
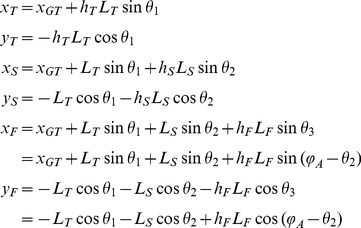
(3)where *x_GT_* is the a-p displacement of the GT marker, *h_S_*, *h_T_* and *h_F_* are the respective longitudinal centre-of-mass positions in percentage of segment length, and *φ_A_* is the ankle angle. *θ_1_* and *θ_2_* are the elevation angles (generalized coordinates) in the reference frame of the horizontal exoskeleton. The segments' inertia parameters of the limbs were estimated on the basis of adjusted regression equations for anthropometric data [40].

In Lagrange's formulation, the kinetic energy *E* of the system is:
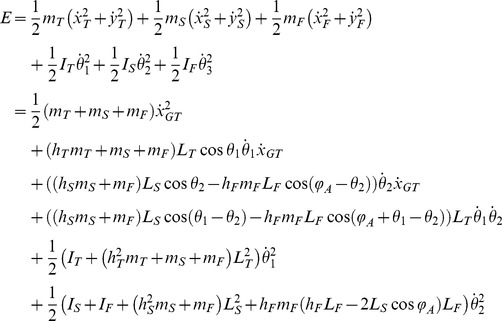
(4)Potential energy *U*, Rayleigh dissipation function *D* (to account for viscosity), and generalized forces *Q_1_* and *Q_2_* are respectively:
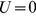
(5)




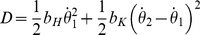
(6)




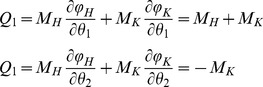
(7)where and *φ_H_* and *φ_K_* are the hip and knee joint angles, respectively.

The motion of the lower limb was derived by solving the Lagrange's equations:
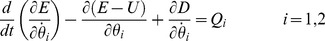
(8)A fourth-order Runge-Kutta method was used to solve eq. 8 with the initial conditions corresponding to the initial static posture (*θ_1_* and *θ_2_* angles) with *M_H_* = 0; *M_K_* = 0; 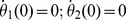.

### Statistics

Descriptive statistics included means ± SD of the mean. Shapiro-Wilk test was used to verify the normality distribution of data. Repeated Measures (RM) ANOVA was used to compare means between different conditions of the main protocol and to evaluate the effect of different speeds for hand-walking in the a-p direction. Post-hoc tests and multiple comparisons analysis were performed by means of Tukey HSD (Honestly Significant Difference) test. The level of statistical significance was set at 0.05. Raw data are available on request from the authors.

### Data availability

The authors make freely available any materials and information described in this paper that may be reasonably requested by others for the purpose of academic, non-commercial research. Please contact f.syloslabini@hsantalucia.it or y.ivanenko@hsantalucia.it.

## Results

### Pilot experiments

We tested 33 participants to assess the percentage of “responsive” subjects. To this end, participants lying in the unloading exoskeleton were asked to rhythmically displace their hands on the overhead treadmill in the antero-posterior direction. To reduce the attention level paid to the locomotor task, participants were always asked to carry out mental arithmetic (counting down out aloud by 7′s) throughout a trial. We found that this type of hand-walking elicited automatic leg movements with a foot excursion L>10 cm in 58% of the subjects (19/33, Fig. 1C). In these responsive subjects, the evoked leg movements were rhythmic, alternating between the left and right leg, and persisted as long as the subject continued to hand-walk. In the remaining subjects of our sample, hand-walking elicited small (L<10 cm) or no detectable movements of the legs.

When interviewed at the end of the experiment, many responsive subjects seemed unaware of having moved their legs during hand-walking. It should be stressed that, because of the posture during hand-walking, view of the lower limbs was essentially prevented. In a few anecdotic cases, when the attention of the subject was directed by the experimenter to the presence of leg movements during the task, the subject appeared surprised and declared that the legs were moving “by themselves”. Nevertheless, if asked to suppress the automatic leg movements, the subject was generally able to do so voluntarily.

### Quantitative assessment of leg movements evoked by hand-walking

We performed in-depth experiments with detailed kinematic and EMG data recording in 9 of the responsive subjects. In these subjects, we found that leg movements were systematically elicited by arm movements over repeated tests, with no sign of adaptation, at least over observation epochs which did not fatigue the subjects (1–2 min). The onset of leg movements always followed that of arm movements, with a time delay that was highly variable across subjects and conditions (Fig. 2). Although there was some initial movement (possibly due to mechanical transmission, see below) of the leg segments roughly at the onset of hand-walking in each responsive case, the delay of a sustained oscillatory leg movement relative to the start of hand movements varied between 0.07 and 30 s in these subjects (on average 4.05±7.04 s). The longest delays tended to occur at the lowest speed of hand-walking (0.5 m/s), but in general the delay did not depend systematically on speed. Thus, the two subjects tested at 1 m/s in Fig. 2 had quite different delays. In most cases (34/45 trials), the delays were less than 3 s. The results presented below refer to the steady-state of evoked leg responses, typically the last 15–30 s of each trial during hand-walking in the a-p direction, hand-walking at 2 km/h in the p-a direction and arm air-stepping.

Another essential feature of locomotor movements is the direction of stepping. We verified whether the leg movements exhibit backward stepping with the reversal of the arm movements. The analysis showed that the foot trajectory area was positive during both a-p (1.5±2.1 cm^2^/cm, range 0÷5.6 cm^2^/cm) and p-a (1.3±2.7 cm^2^/cm, range 0÷8.9 cm^2^/cm) directions of hand-walking at 2 km/h. Thus, the evoked rhythmic movements were predominantly forward independent of the direction of hand-walking.

**Figure 4:**
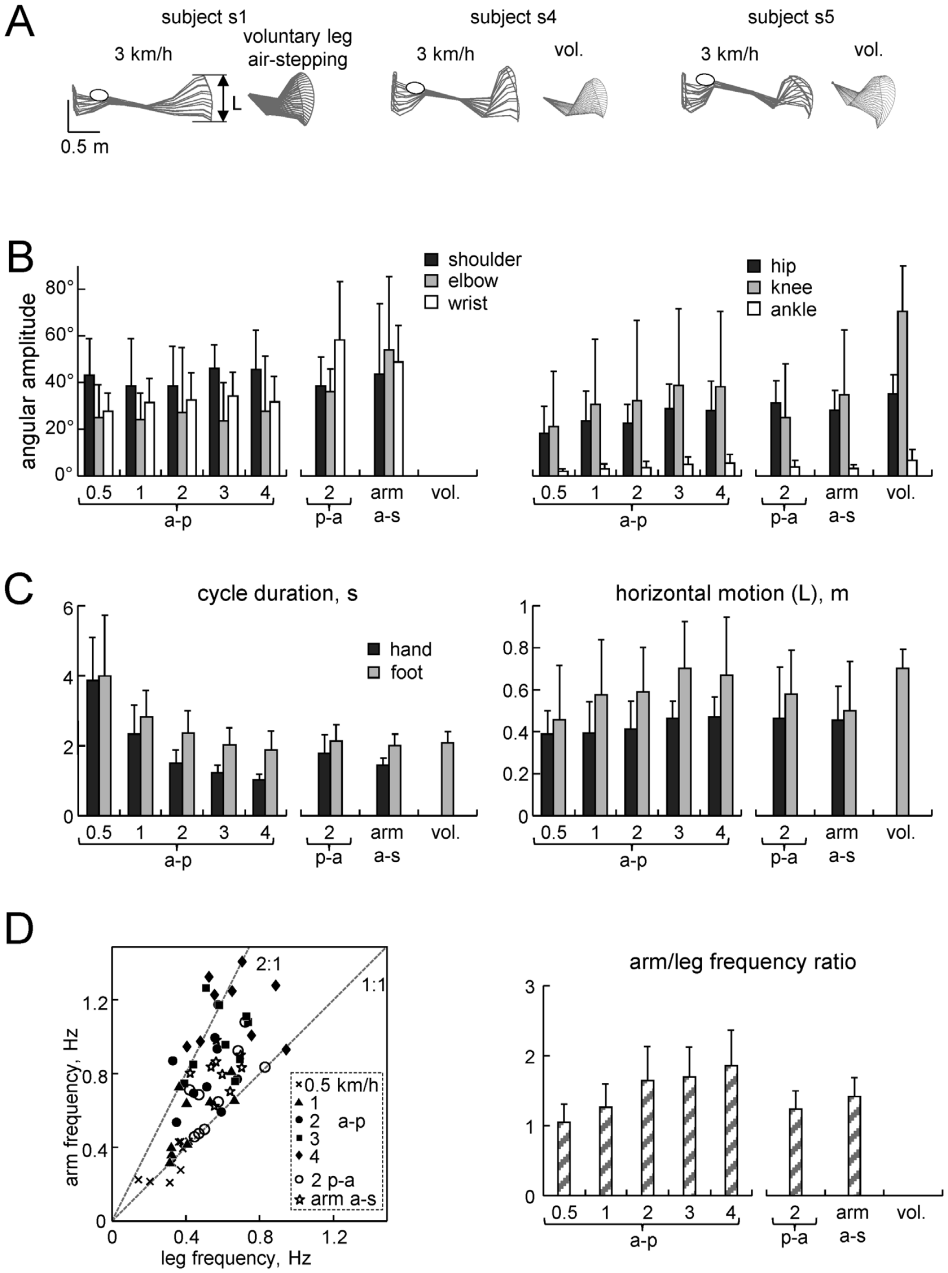
General gait parameters. A – stick diagrams of left arm and leg movements in three different subjects during hand-walking at 3 km/h in the a-p direction (left) and voluntary (vol.) air stepping (right). B – mean (+SD, n = 9) arm and leg joint angular amplitudes across different hand-walking speeds in the a-p direction (left) and different conditions of the main protocol: hand-walking at 2 km/h in the p-a direction, arm air-stepping (arm a-s) and voluntary leg air-stepping. C – cycle duration and a-p foot excursion. D – frequency relation between arm and leg movements. In scatter plots each point illustrates the arm or leg movement frequency during different conditions for each single participant. The dotted lines indicate integer arm:leg frequency ratios (1∶1 and 2∶1). Bars in the right panel represent mean (+SD) arm/leg frequency ratios over all conditions.

Figure 4 presents the general kinematic parameters for both arm and leg (evoked) movements. The angular motion of shoulder and elbow joints was not significantly different (F(2,16) = 0.227, p = 0.80 for shoulder and F(2,16) = 2.45, p = 0.12 for elbow, RM-ANOVA) across different hand-walking conditions (a-p at 2 km/h, p-a at 2 km/h and arm air-stepping). The wrist joint angular amplitude was significantly larger (p = 0.035, Tukey HSD) during hand-walking in the p-a direction than during hand-walking in the a-p direction (Fig. 4B, left panels). The relative angular motion in the hip and knee joints during evoked, automatic leg movements were variable across subjects (Fig. 4A). On average, they were ∼30° smaller in the knee joint (Fig. 4B, p<0.05, Tukey HSD) relative to those during voluntary leg air-stepping (Fig. 4B, right panels).

Ankle joint angular motion was quite small during evoked leg movements and comparable to that recorded during voluntary air-stepping (p≥0.11, Tukey HSD). The horizontal foot excursion and the cycle duration of evoked leg movements were comparable with those of voluntary leg air stepping (Fig. 4C, p≥0.052, Tukey HSD). The cycle duration of both arm and leg oscillations decreased monotonically with speed (F(4,32) = 54.4, p<0.00001 for arms and F(4,32) = 8.30, p = 0.00027 for legs, RM-ANOVA, Fig. 4C).

However, in contrast with what usually occurs in upright walking [5], [41], the frequency ratio between arm and leg movements differed from 1 in most cases during hand-walking, and depended significantly on the speed of hand-walking (F(4,32) = 11.8, p<0.00001). Indeed, when the frequency of arm movements are plotted against that of leg movements (Fig. 4D), the data points for hand-walking in all conditions fall between the 1∶1 and 2∶1 regression lines indicating that the frequency of leg movements tended to be lower than that of arm movements.

### EMG patterns during hand-walking

**Figure 5:**
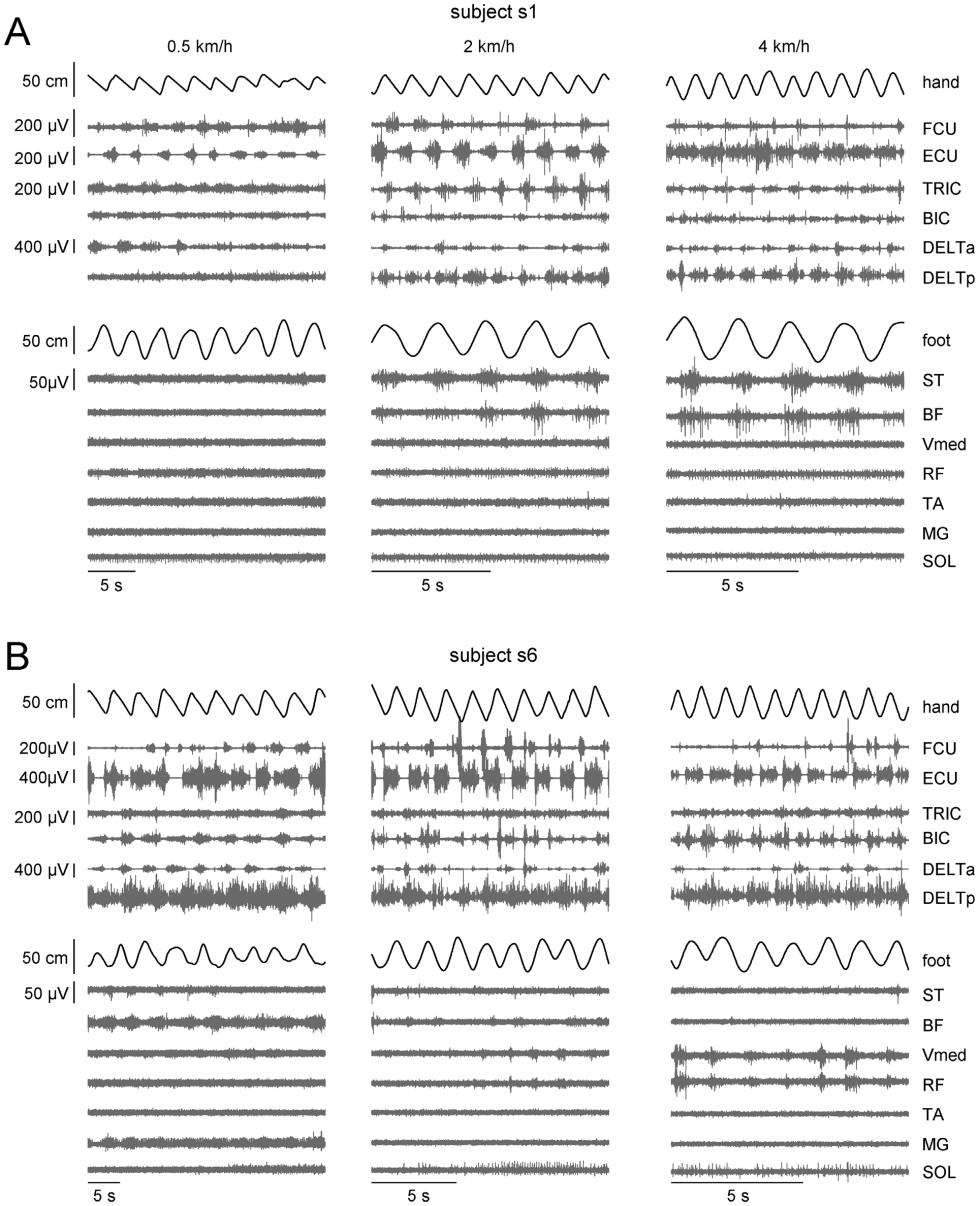
EMG patterns in two representative subjects for different hand-walking speeds. From *top* to *bottom*: anterior-posterior displacement of the left hand, EMG activity of 6 muscles of the left arm, a-p displacement of the left foot and EMG activity of 7 muscles of the left leg. Note non-linear changes in the EMG activity with speed: subject 1 (A) showed an increment in hamstring muscle activity while subject 2 (B) showed a decrease in hamstring and an increase in quadriceps activity. Note also the absence of noticeable activity in distal leg muscles.

**Figure 6:**
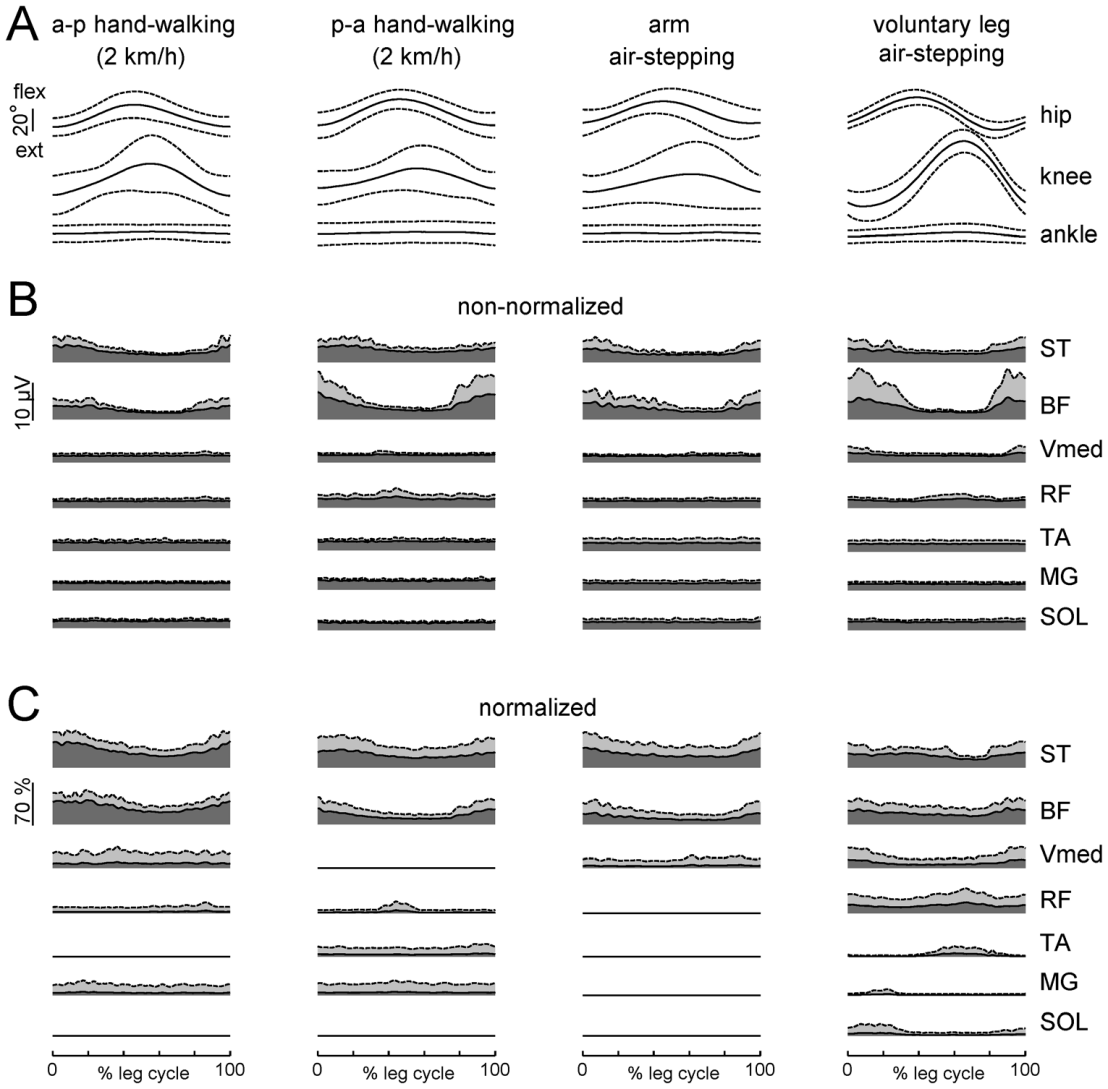
Ensemble averaged (across subjects) kinematic and EMG patterns during hand-walking. From *left* to *right*: hand walking at 2 km/h in a-p direction, p-a direction, arm air-stepping and voluntary leg air-stepping. From *top* to *bottom*: joint angles (mean±SD, n = 9) (A), non-normalized (B) and normalized (C) EMG envelopes (black lines indicate the mean and dashed lines indicate mean+SD). Patterns are plotted versus normalized leg cycle. Note similar timing of hamstring muscle activity (around the onset of the leg cycle) across all conditions.

**Figure 7:**
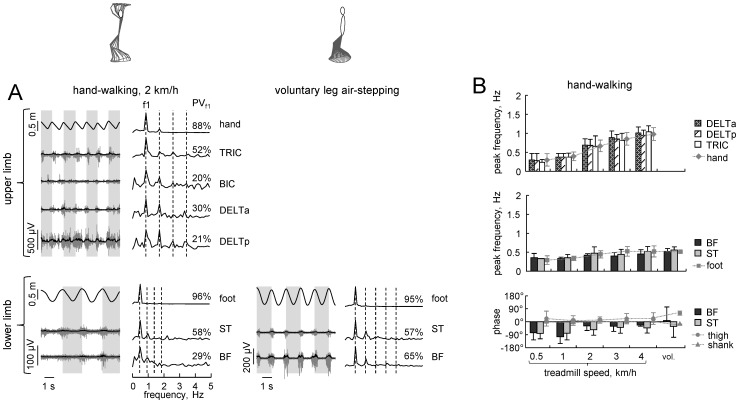
Coordination of arm and leg movements. A – arm and leg kinematics and EMGs in one representative participants performing hand-walking at 2 km/h and voluntary air-stepping. The left portion of each panel shows the oscillations of left upper and lower limbs accompanied by respective EMG activity of some representative muscles. Grey areas demarcate single limb cycles defined separately for arms and legs as the time between two consecutive maxima in the a-p displacement of the respective endpoint. The right portion of each panel displays the Fourier spectra derived from the signals on their left. Dashed lines show the position of the first five multiples of the fundamental frequency (f1) whose percentage of variance explained is displayed (PV_f1_). B – mean (±SD, n = 9) peak frequencies of EMGs and corresponding arm (upper plot) and leg (middle plot) kinematic patterns for hand-walking at different speeds and for voluntary leg air-stepping (vol.). Lower plot represent the phase (relative to the onset of leg cycle) of the first harmonic for leg EMGs and thigh and shank elevation angles (zero corresponds to the cosine function with zero time shift). Only EMGs with peak activity greater than 2 µV and PV_f1_>20% were included in this analysis.

Hand-walking was generally associated with a low level of EMG activity of leg muscles, consistent with the unloaded conditions of a horizontal posture and with limited movements at the ankle joint (Figs. 2,5,6). Indeed, overall EMG activity was low even during voluntary air stepping (see Figs. 6,7), which also involved unloading and limited ankle movements (see also [42]). Moreover, there was inter-subject variability in the modulation patterns of leg muscle activity associated with hand-walking (compare the two subjects of Fig. 5). EMG tended to be modulated rhythmically in-phase with the leg movements in hamstring muscles (ST and BF) most frequently, and in other lower limb muscles more sporadically. Thus, subject s6 in Fig. 5B showed appreciable modulation of vastus medialis (Vmed) and rectus femoris (RF), while subject s2 in Fig. 2 showed modulation of gastrocnemius medialis (MG). Also the changes of EMG activity with speed were somewhat subject-dependent. For instance, the activity of hamstring muscles increased with speed in subject s1 in Fig. 5A, while it decreased with speed in subject s6 in Fig. 5B (and the activity of Vmed and RF increased with speed in this subject).

Figure 6 compares the leg kinematic and EMG patterns, ensemble averaged over all subjects during hand-walking (a-p and p-a at 2 km/h), arm air-stepping and voluntary air-stepping. Notably the pattern of modulation of hamstring muscles in hand-walking was similar to that in voluntary leg air-stepping, with activity bursts around the end of swing and beginning of stance in both conditions (see Fig. 6). Also leg kinematics in hand-walking resembled that in voluntary air-stepping, both conditions involving much larger changes of hip and knee angles than ankle angle. The angular excursion of leg joints was low during air stepping (both evoked and voluntary) especially in the ankle joint (Fig. 6).

### Coordination of arm and leg movements

To characterize quantitatively the coupling between arm and leg movements, we computed the magnitude and phase of the Fourier transform of kinematic and EMG variables (Fig. 7). For leg movements, we analyzed only the activity of hamstring (BF and ST) muscles, because these were the muscles more consistently modulated during hand-walking and voluntary air stepping among those recorded.

Figure 7 illustrates kinematics and EMGs in one representative participant performing hand-walking in the a-p direction at 2 km/h and voluntary air-stepping. In particular, these examples show that, in contrast to upright walking [2], the arm and leg oscillation frequencies during hand-walking were not locked between each other in a 1∶1 relationship. These relationships between arm and leg movements were confirmed by analyzing the EMG activity of proximal arm and leg muscles, even though the percent of variance explained by the fundamental harmonic was typically smaller for the EMG profiles compared to kinematics for both hand-walking and voluntary leg air-stepping (Fig. 7A).

The peak frequency of modulation of EMGs was tightly correlated with that of the corresponding limb, the arm for arm muscles, and the leg for leg muscles (Fig. 7B, upper and middle panels). Fourier analysis also confirmed the previous qualitative observation (Fig. 6) that the phase of BF and ST muscle activity for hand-walking (Fig. 7B, lower panel) was roughly similar to that for upright walking (around the end of swing and beginning of stance [43]).

When the ratio between arm and leg movements was ∼1 (in the range 0.95÷1.05, 13 trials total across all conditions), ipsilateral phase lag (eq. 2) between upper and lower limbs was 34±8% (range 22÷47), thus exhibiting a more ‘diagonal’ gait (IPL close to 50%) though in the four out of 13 trials participants showed no limb paring (IPL = 22–28%). ‘Lateral’ gait (IPL∼0%) was never observed.

### Automaticity assessment

According to the literature [44], [45], evidence that a task is automatic and carried out with minimal attention is provided by the fact that a secondary task is performed with little interference. To evaluate the extent of task interference, in four subjects we compared the performance of mental arithmetic (the number of errors and rate of counting) during resting periods and during hand walking. Neither the percent of errors (5±2% and 7±4% during rest and hand-walking, respectively) nor the rate of counting (18.8±7.5 min^−1^ and 15.2±5.4 min^−1^, respectively) were significantly different in these conditions (p = 0.18 and p = 0.05, paired t-tests), although the performance of mental arithmetic was slightly worse during hand-walking. These findings showed limited interference between the two tasks, thus supporting the hypothesis about automaticity of the leg movements evoked during hand-walking [44], [45]. It should also be noticed that mental arithmetic tends to minimize attention of the subjects to leg movements.

### Direction-specific effect of hand-walking

**Figure 8:**
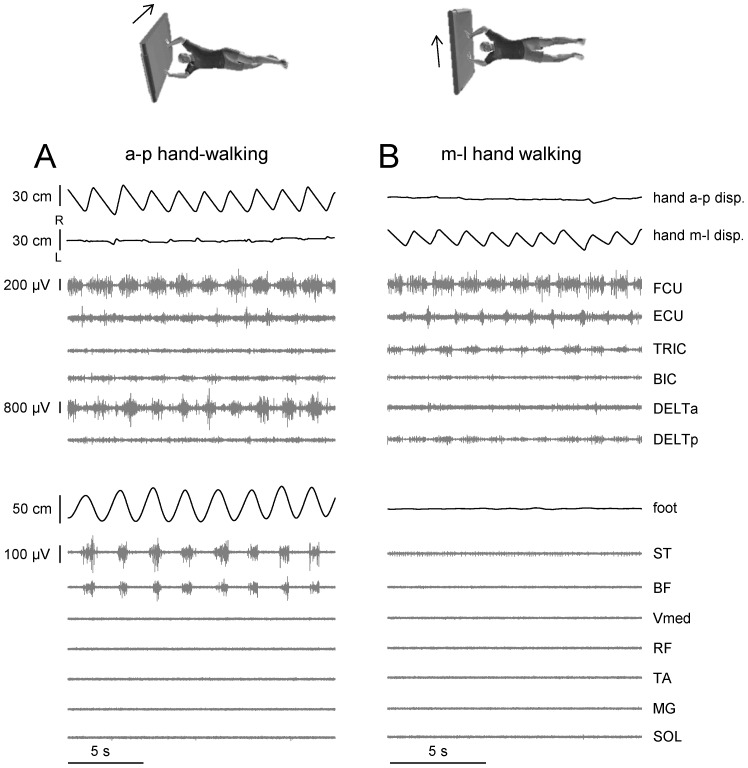
Hand-walking direction specificity of evoked leg movements. An example of leg movements during hand walking in anterior-posterior (a-p, A) and medio-lateral (m-l, B) directions in one representative subject (s9). Similar format as in Fig. 5. R – right, L – left. Note minute if any leg movements during hand walking in the m-l direction (B).

Another question is whether the effects are direction-specific or caused by a generic increase of excitability of the spinal locomotor circuitry due to arm muscle contractions. For instance, it is known that the Jendrassik maneuver facilitates leg air-stepping [33]. Specifically, we tested whether automatic leg movements can be elicited by arm movements performed in a direction different from that of normal walk. To this end, we asked four subjects to perform hand-walking in either anterior-posterior or medio-lateral directions at the same speed (Fig. 8). Hand excursions in the direction of treadmill belt movement were not significantly different between the two conditions (28±6 cm vs. 29±8 cm respectively, p = 0.39, paired t-test, even though the example in Fig. 8 shows slightly smaller hand excursions in the m-l direction). However evoked leg movements were principally observed during a-p hand-walking (foot displacements were 49±27 cm vs. 4±3 cm, p = 0.021, paired t-test). Furthermore, no significant differences were found in the performance of the mental arithmetic task as quantified by the percent of errors and counting rate between hand-walking in a-p and m-l directions (p>0.32 for both parameters, paired t-tests), consistent with a similar level of automaticity for hand-walking in the two directions.

### Effects of blocking leg movements

**Figure 9:**
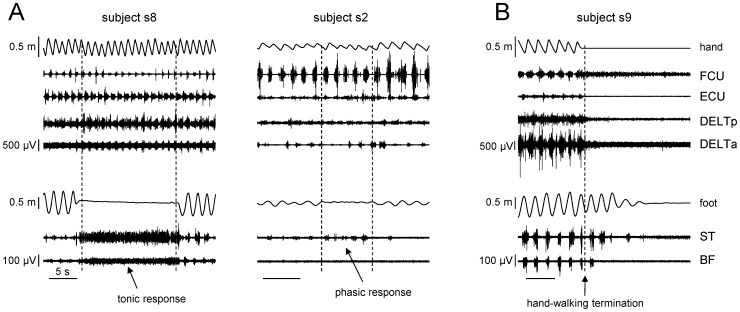
Effect of transient leg block and termination of hand-walking. A – Upper and lower limb kinematics and EMG activity in two subjects during the transient block of the legs performed manually by an experimenter. Dashed lines delimit the period of transient leg block: the cessation and onset of foot displacements. One subject (s8) exhibited a tonic response in leg muscles during the block while the other (s2) showed a phasic response. All subjects restored non-voluntary air stepping movements after the release of the legs. B – continuation of leg movements after terminating hand-walking. Note EMG activity of ST and BF muscles associated with a few post-cycles of foot movements.

To further investigate the nature of evoked leg movements, we blocked leg movements manually in six subjects. Figure 9 illustrates examples of the evoked responses in four subjects: dashed lines delimit the period of transient leg block (interruption and resumption of foot displacements). In most trials (27/42, 64%), we observed hamstring (ST, BF) muscle EMG activity in response to this maneuver. We did not observe any noticeable EMG activity in other leg muscles. In 17 trials, subjects exhibited a tonic response consisting of a persistent activation during the block (see subject s8 in Fig. 9A), while in 10 trials we observed a rhythmic response consisting of bursting episodes at about 0.6 Hz (subject s2 in Fig. 9A). Table 2 contains the number of trials with tonic and phasic responses in each subject. In the remaining 15 trials, there was no evident rhythmic or tonic hamstring muscle EMG activity. All subjects resumed automatic leg movements upon release of the legs while they continued to hand-walk (Fig. 9A).

**Table 2:** Number of trials in each subject with tonic and phasic responses in the hamstring muscle to the transient block of the legs.

		number of trials		
subject	total number of trials	tonic response	phasic response	no response
s2	13	1/13	5/13	7/13
s5	4	1/4	3/4	0/4
s6	7	7/7	0/7	0/7
s7	6	1/6	2/6	3/6
s8	3	3/3	0/3	0/3
s9	9	4/9	0/9	5/9
total	42	17/42 (40%)	10/42 (24%)	15/42 (36%)

### After-effect of hand-walking termination

We also tested the persistence of leg movements and/or EMG activity following hand-walking termination (Table 1, three trials at 1 km/h in the a-p direction for each subject). Persistent leg movements with corresponding hamstring muscle activity were observed following the termination of arm movements (Fig. 9B). The delay of the termination of oscillatory leg movements relative to the end of hand movements varied between 0.6 and 46 s. In most cases (9/12 trials), the delay was longer than 3 s (on average 10.05 s).

### Effects of trunk oscillations on leg motion

The potential mechanical effects of trunk oscillations on leg motion were assessed using both biomechanical modeling and the effect of externally imposed trunk movements. The results of modeling are presented in Fig. 3. On averaged, horizontal (peak-to-peak) foot displacements predicted by the model using actual hip movements during hand-walking as an input were 8±7 cm (all speeds were pooled together, Fig. 3C).

To estimate directly the effects of trunk oscillations on leg movements (Table 1) the experimenter moved the trunk of the subject back and forth, trying to mimic the hip oscillations associated with hand-walking. The resulting average displacement of the hips was 5.45±1.41 cm, as compared with an average displacement of 3.53±2.49 cm associated with hand-walking (Fig. 3C, left panel). Importantly, we found that the amplitude (peak-to-peak) of foot (MP marker) displacements caused by passive hip movements was relatively small (between 2 and 10 cm, on average 7.30±3.41 cm). Moreover, in contrast with the leg movements evoked by hand-walking, we did not observe any appreciable EMG activity in leg muscles with passive hip movements (Fig. 3B). We also found that the amplitude of horizontal foot displacement predicted by the model was not significantly different from that observed experimentally with passive hip movements (Fig. 3B and C, p = 0.67, Tukey HSD).

In sum, foot movements evoked by hand-walking were significantly larger than both those experimentally measured with passive hip motion and those predicted by the biomechanical model (for both comparisons, p = 0.00017 Tukey HSD). The same was also true for joint angle excursions (Fig. 3C). Foot excursions induced by passive hip displacements did not exceed 10 cm (Fig. 3C). Therefore, we used this limit (L = 10 cm) as the lower threshold of leg movements associated with hand-walking to identify “responsive” subjects from the pilot experiments (Fig. 1C).

## Discussion

We found that moving the arms rhythmically on an overhead treadmill, as in hand-walking, often elicited automatic, alternating movements of the legs in a significant proportion of tested subjects. These leg movements showed some similarities to those of voluntary air-stepping and upright locomotion. Thus, the timing of hamstring EMG activity relative to the gait cycle was similar across all three conditions. Moreover, as in normal walking, the frequency of leg movements increased with increasing frequency of arm movements during hand-walking. However, the arm/leg frequency ratio tended to become greater than 1 with increasing treadmill speed, in contrast with the fixed ratio of 1 of normal walking. The coupling between the activity of cervical motoneurons underlying hand-walking and the activity of lumbosacral motoneurons underlying leg movements was presumably indirect, delayed and asynchronous, at least under our experimental conditions. Below we discuss the results in the context of possible functional linkages between cervical and lumbar networks that could be responsible for the observations.

### Methodological considerations

Our setup allowed relatively unconstrained leg motion in a gravity equipotential plane, while hand-walking was performed at controlled speeds. Despite its advantages for investigating rhythmogenesis, the setup has limitations. First, the lying position of the subject results in asymmetric vestibular and tactile stimuli to the body and in some arm muscle activity against a gravity force acting perpendicularly to the arm's long axis. However, it is unlikely that these position-related factors had significant effects on the general stepping characteristics. Indeed, it has previously been shown that walking in this reclined setup is very similar to that with a vertical body weight support [35], [36]. Also, we do not think that leg movements represent a strategy used to facilitate the ‘vertical’ reach by the arms to the treadmill in late ‘swing’ of the arm cycle, because leg motion was not observed during medio-lateral hand-walking (Fig. 8). Finally, rhythmic leg movements were consistent across different conditions including different postures of the arms and the presence or absence of ‘ground reaction’ forces for hand-walking (arm air-stepping, Figs. 4,6).

Second, arm positioning above the head corresponds to ‘walking on trees’ [46] or to a swimming-like arm orientation [2] rather than to arm swing during overground locomotion. Nevertheless, a-p shoulder oscillations during hand-walking (∼7 cm) were comparable to those during normal walking (∼5–10 cm [47]). Also, human bipedalism is often thought to have evolved from a quadrupedal precursor (either terrestrial or arboreal [26], [46]). One of the distinctive aspects of primate quadrupedal walking is the use of diagonal couplets of interlimb timing [16], [48]–[50] that we also observed in our experiments when the ratio between arm and leg frequencies was 1∶1.

Third, we studied the effects of upper limb movements on lower limbs movements when both upper and lower limbs were not weight-bearing. In these conditions, the ankle joint was typically not involved (Figs. 4B) likely due to the absence of loading forces, consistent with previous studies using tonic sensory stimulations (by means of muscle vibration or nerve electrical stimulation) to evoke leg air-stepping movements [32], [33]. Sensory feedback makes a substantial contribution to the activation of distal muscles during locomotion [51], [52], and the pattern generation circuitry in the sacral cord [53] could possibly be inactivated when the input from the support surface is lacking. The cycle duration of evoked leg oscillations (on average 2–4 s, Fig. 4C) was longer than during upright walking (1.1–1.6 s, depending on speed [54]), consistent with the effects of gravity on the pendulum-like behavior of the limbs and with the idea that the locomotor controller takes advantage of and adapts to the passive dynamics of the multi-joint system [5], [36]. We used air-stepping as a model for investigating rhythmogenesis in humans since its manifestation is largely facilitated by the absence of external resistance and it engages intact sensory inputs [33], [34]. Moreover, it is known that many features of quadrupedal arm-leg coordination are conserved across different locomotor tasks in humans [16], [55] including a reciprocal pattern of influences between the coordination of reaching and walking [56] or quadrupedal limb coordination during obstacle avoidance [57].

Finally, a mechanical transmission of arm movements and associated trunk torsion to the legs cannot be excluded, considering the low resistance of the exoskeleton. However, using externally imposed trunk movements and biomechanical modeling (see *Effects of trunk oscillations on leg motion* in the Results), we showed that passive hip displacements roughly comparable to those recorded during hand-walking determined leg movements much smaller than those associated with hand-walking and with no detectable modulation of EMG activity in leg muscles. Instead, such EMG modulation was present during hand-walking (Figs. 2,5,6) and often continued even when the leg movements were transiently blocked by the experimenter (Fig. 9A) or following the termination of arm movements (Fig. 9B). The bulk of the evidence, therefore, points to a predominantly active (neural) rather than passive (mechanical) nature of the leg movements evoked by hand-walking.

### Putative mechanisms

It is unlikely that the leg movements were generated voluntarily during hand-walking: the subjects were always involved in mental arithmetic, and, when asked, they appeared unaware of the legs movements (which they could not see due to the posture). Indeed, it is generally accepted that automatic movements are performed without attention being clearly directed toward the details of the movement [58]. In addition, performance of mental arithmetic (percent of errors and rate of counting) was little affected by hand-walking, suggesting minimal or no interference with the motor task and thus the automaticity [44], [45] of leg movements.

Although the leg responses were presumably automatic, they were not stereotyped. Thus, the delay in the onset of leg oscillations relative to arm oscillations was variable (typically about 1–2 s, but sometimes much longer), as was the relationship between the frequency of leg oscillations and that of arm oscillations (Fig. 4,7). Just as the voluntary air stepping, also hand-walking was generally associated with a low level of EMG activity of leg muscles, due to the unloaded conditions. The hamstring muscles were the leg muscles more consistently activated in a rhythmic fashion, but quadriceps and gastrocnemius were also modulated rhythmically in some subjects (Fig. 2,5,6). This can be explained by the important contribution of stretch reflexes in the hamstring muscle (especially at end swing) in the context of a “passive” contribution [59]. Moreover a limited activation of more distal muscles can be interpreted in terms of their stronger dependency on sensory feedback related to limb loading as compared with proximal muscles (in the context of “active” contribution from central sources) [51], [52]. When the leg movements were transiently blocked by the experimenter (Table 2), or following the termination of arm movements (Fig. 9), in different trials we could observe one of 3 different responses in the EMG activity of the previously active muscles: 1) rhythmic activity, 2) tonic activity, or 3) no detectable activity. The persistence of leg rhythmic activity (Fig. 9A,B) further supports an active (neural) rather than passive (mechanical) nature of the leg movements evoked by hand-walking and thus points to activation of pattern generating circuits. Instead, the presence of tonic activity may depend on the interrelation between muscle tone and locomotion. There are many examples of such interrelation. For instance, muscle activity can be prolonged when the motion of the limb is interrupted in the swing or stance phase and the alternating bursts may be replaced by tonic activity when the limb is held stationary [60]–[62]. Moreover, epidural stimulation at the L5 spinal level in decerebrated cats initially induces tonic activity in hindlimb muscles that changes to locomotor-like activity after 5–7 s of stimulation [34]. Also, initiation of brainstem-evoked locomotion is generally accompanied by an increase in postural muscle tone [63]. Finally, different forms of human locomotion may be associated with specific muscle tone [64]. Large inter-individual differences in humans (Fig. 1C,2,9) in the delay and responsiveness of spinal pattern generation circuitry to its activation have also been reported in previous studies [32]–[34].

Whatever the exact mechanism of the observed phenomenon, these variable features suggest that signals related to arm movements did not directly entrain the motor commands to leg muscles, but triggered responses that depended on sensory feedback and the state of the lumbosacral locomotor circuitry [65]. One possible route for these trigger signals is through the intrinsic spinal pathways linking cervical to lumbosacral neurons. The best known of such connections is represented by the long descending propriospinal neurons which have been demonstrated in humans [14]. However, considering the latency of the leg responses relative to arm oscillations, supraspinal contributions cannot be excluded.

Both descending and ascending connections between cervical and lumbosacral CPGs have been described in quadrupedal mammals. In these animals, the inter-limb coupling is much stronger than in humans, but the functional state of these connections is task and context dependent [18]. Our results demonstrate that, like in quadrupedal animals [19], [29]–[31], rhythmic upper limb movements can initiate lower limb stepping in bipedal humans. Furthermore, both in cats (see Fig. 2 in [31]) and humans (Fig. 4,7), forelimb-assisted hindlimb stepping is often characterized by a non-integer ratio between forelimb and hindlimb movements. Moreover, it is worth stressing that the observed phenomenon is specifically attributed to cyclic arm movements. For instance, a strong isometric contraction of arm muscles (as in the Jendrassik maneuver) may increase the excitability of reflex pathways [66], [67], but it does not evoke leg oscillations [33]. Also, medio-lateral rhythmic arm movements are not effective in triggering leg air-stepping (Fig. 8), supporting the hypothesis that the mechanisms underlying this phenomenon are attributable to direction-specific arm movements rather than to a generic increase of excitability of the leg motor circuitry due to arm muscle contractions. Indeed, arm swinging normally occurs in the a-p direction during normal locomotion.

## Conclusions

The present results reinforce the idea that in humans there exists a neural coupling between arm and legs in humans [3], [22]. In addition to the physiological relevance, the present findings may have clinical implications. Indeed, rhythmic arm movements could be effective in the rehabilitation of lower limb paresis. In general, a better understanding of both bottom-up and top-down pathways coordinating movement of the four limbs [18] could have important implications for gait rehabilitation in Parkinson disease [42], [68], spinal cord injury [22], stroke [69], cerebral palsy [70] and other neurological injuries which disrupt interlimb coordination.
